# The BUME method: a new rapid and simple chloroform-free method for total lipid extraction of animal tissue

**DOI:** 10.1038/srep27688

**Published:** 2016-06-10

**Authors:** Lars Löfgren, Gun-Britt Forsberg, Marcus Ståhlman

**Affiliations:** 1 Cardiovascular and Metabolic Diseases, Innovative Medicines and Early Development Biotech Unit, AstraZeneca Gothenburg, Sweden; 2 Wallenberg Laboratory, Sahlgrenska Academy at University of Gothenburg, Gothenburg, Sweden

## Abstract

In this study we present a simple and rapid method for tissue lipid extraction. Snap-frozen tissue (15–150 mg) is collected in 2 ml homogenization tubes. 500 μl BUME mixture (butanol:methanol [3:1]) is added and automated homogenization of up to 24 frozen samples at a time in less than 60 seconds is performed, followed by a 5-minute single-phase extraction. After the addition of 500 μl heptane:ethyl acetate (3:1) and 500 μl 1% acetic acid a 5-minute two-phase extraction is performed. Lipids are recovered from the upper phase by automated liquid handling using a standard 96-tip robot. A second two-phase extraction is performed using 500 μl heptane:ethyl acetate (3:1). Validation of the method showed that the extraction recoveries for the investigated lipids, which included sterols, glycerolipids, glycerophospholipids and sphingolipids were similar or better than for the Folch method. We also applied the method for lipid extraction of liver and heart and compared the lipid species profiles with profiles generated after Folch and MTBE extraction. We conclude that the BUME method is superior to the Folch method in terms of simplicity, through-put, automation, solvent consumption, economy, health and environment yet delivering lipid recoveries fully comparable to or better than the Folch method.

Lipids are a heterogeneous group of molecules with enormous structural diversity. It is therefore not surprising that lipids are involved in many biological processes and are thought to be part of the etiology of several common diseases such as type 2 diabetes, Alzheimer’s disease and cancer^1^,^2^. From an analytical point of view, the immense combinatorial and structural diversity of lipids have led to technical challenges associated with the determination of unique molecular species among thousands of different lipid isoforms. In recent years however, lipid research has seen a renaissance mainly driven by new advances in mass spectrometry technology. Using mass spectrometry it is now possible to generate quantitative data from hundreds of lipid species from small amounts of sample material, an approach sometimes referred to as lipidomics^3^,^4^,^5^. In addition, the development of ultra-performance liquid chromatography (UPLC) has reduced the analytical runtime, which means that hundreds of samples can be run automatically and unsupervised within 24 hours.

An important part of the workflow of lipid analysis is the extraction procedure. This step purifies the lipids and removes unwanted and potentially interfering substances such as proteins, carbohydrates and other polar metabolites. While there has been an impressive development of automated and high-throughput oriented analytical methods, the extraction procedure is still often performed manually with traditional methods. Two of the most commonly used methods are the Folch^6^ and Bligh and Dyer^7^ methods. These methods were published over 50 years ago and are based on chloroform, which makes these methods highly efficient in extracting lipids with a wide range of polarity. However, there are several drawbacks with these methods besides the use of chloroform, which is a known carcinogen. One major draw-back is that the lipids need to be retrieved from the bottom fraction after the two-phase separation. This means that the upper aqueous phase and the intermedia, containing precipitate and insoluble material, need to be penetrated. This might lead to contamination of the lipid extract, which in turn might compromise the analysis. Furthermore, this risk of contamination and plugging of the tips used for lipid extract transfer increase when using automated and unsupervised protocols for these kind of lipid extraction procedures.

To overcome the drawbacks of a lower organic phase and to facilitate automation alternative methods have been developed. Matyash *et al*. recently showed that a lipid-enriched upper phase can be formed by using methyl-tert-butyl ether (MTBE) in combination with methanol and water^8^. Furthermore, the method showed high recoveries for all investigated lipids when compared to the gold standard Folch method. Furthermore, our group recently published the fully automated chloroform-free BUME method for total lipid extraction of biofluids using a standard pipetting 96-well robot^9^. We have now extended and validated this method for 15–150 mg tissue samples. The method, which is based on butanol and methanol (BUME) for the initial one-phase extraction, is an “all-in-one-tube” method performed in 2 ml polypropylene tubes containing ceramic beads for the initial homogenization step. Besides the benefit of being able to perform the complete process of sample collection, storage, homogenization and extraction procedure in the same small sample collection tube, the chosen solvent system also results in a lipid-enriched upper phase. This enables the use of a pipetting robot for automatic transfer of lipid extracts without the risk of contamination by the water and intermedia phase. The automated transfer also minimizes the risk of errors and relieves strains on neck and shoulders. By using the developed method, 96 tissue samples can be extracted in 4 hours moving sample preparation into the high-throughput workflows, which are fundamental in the field of lipidomics.

## Methods

### Standards and Chemicals

Butanol, heptane, ethyl acetate, MTBE, methanol, chloroform and acetonitrile were all of HPLC grade and attained from Rathburn Chemicals Ltd (Walkburn, UK). Isopropanol was from Acros Organics (Pittsburgh, PA, USA) and acetic acid was from Merck (Darmstadt, Germany). Non-radiolabelled lipid standards were from Avanti lipids (Alabaster, AL, USA) with the exception of d_6_-triglyceride (TG, tripalmitate) and d_5_-cholesteryl ester (CE, oleate), which were from CDN isotopes (Quebec, Canada). Radiolabeled ^14^C phosphatidylcholine (PC, 1-palmitate, 2-linoleate), ^14^C lysophosphatidylcholine (LPC, 1-palmitate) and ^14^C CE (oleate) were from Amersham Biosciences (Little Chalfont Bucks, UK). Radiolabeled ^14^C TG (tripalmitate) and ^14^C diglyceride (DG, dioleate) were from American Radiolabeled Chemicals Inc (St Louis, MO, USA) while the ^14^C free (unesterified) cholesterol (FC), ^3^H sphingomyelin (SM, palmitate), and ^14^C palmitic acid were from Perkin-Elmer life science (Boston, MA, USA). Standard serum Seronorm Lipid (freeze-dried bovine serum) was from SERO AS (Billingstad, Norway).

### Tissue samples

The tissues used in the experiments were collected and immediately snap-frozen in liquid nitrogen. For optimization of homogenization parameters mouse tissue was used. The tissues investigated (about 50 mg) were liver, heart, muscle (red quadriceps), colon and brain.

For the linearity and recovery tests dog liver was used. To attain enough sample material, and also to reduce the natural biological variation between and within tissue samples, several liver pieces were put in liquid nitrogen and grinded to about 3 grams of a fine powder. For the linearity test 15–150 mg was used (n = 40) and for the recovery experiment about 20 mg of powder was used as a matrix (n = 6).

To further compare BUME to the gold standard Folch and the more recent MTBE method, lipids from mouse hearts and mouse liver were extracted using the three procedures (n = 6). Again several pieces of tissue were pulverized in order to minimize the biological variation. Between 20–60 mg tissue was used in all procedures.

Experimental procedures were in accordance with Swedish laws on the use of animals for experimentation and were approved by the Gothenburg Region Ethics Review Committee on Animal Experiments. Ethical application for the Gothenburg region nr: 108–2011 (mice) and 280–2008 (dog).

### Tissue homogenization and BUME extraction

The snap-frozen tissue was weighed at −20 °C into 2 ml reinforced homogenization tubes pre-filled with 2.8 mm zirconium oxide beads (CK-28-R,cat no KT03961-1-007.2) from Bertin Technologies (Montigny-le Bretonneux, France). As a low-cost alternative to these ready-made homogenization tubes, empty reinforced 2-ml tubes (Bertin cat no KT03961-1-403.2) or Sarstedt tubes (item nr: 72.694.007, Sarstedt, Germany) loaded with 6 zirconium oxide beads (3mm) (Retsch, Haan, Germany) were used in our laboratory. During weighing and addition of cold (−20 °C) BUME (butanol:methanol [3:1]) solution, the samples were kept in pre-cooled alumina blocks designed in-house for temperature control during sample handling (Suppl. Fig. 1). These blocks are now commercially available and can be ordered from Bönhult Industriteknik AB (Gothenburg, Sweden). The tissue was homogenized at power 5000 using a Precellys 24 (Bertin technologies) leaving samples to cool on ice between 1–2 repeated 20-second cycles. The homogenization and extraction of up to 2 × 24 tubes simultaneously was then continued at 25 Hz for 5 minutes using a Mixer Mill 301 instrument (Retsch GmbH, Haan, Germany). The automated liquid handling steps in the extraction procedure were performed by a Velocity 11 Bravo pipetting robot (Agilent technologies, Santa Clara, CA, USA). The robot, which works in the 96-well format, can handle 24 homogenization tubes simultaneously, placed in a “24/96” format rack (every second column and row used to allow 24 individual 2-ml tubes in a 96-well foot-print). To allow inspection of the automated liquid handling process, the samples were moved from the alumina rack to shallow “24/96” format PVC racks. It is not necessary to keep the samples cool after the initial single-phase extraction performed at −20° as described above. The lipid extracts in the homogenization tubes were transferred by the Bravo robot into individual 1.2 ml glass vials positioned in the “24/96” format in the 96-well alumina rack.

### Folch and MTBE extraction.

Tissue (powdered heart or liver) was homogenized in 500 μl MeOH using Precellys and Mixer Mill 301 instrument as described above for the BUME protocol. The homogenate was transferred to 15 ml glass tube and lipids were extracted using a modified Folch procedure^6^ or according to the MTBE method^8^.

### Folch method

After transfer of the homogenate to the 15 ml glass tube, the homogenization tube was washed with 500 μl of methanol. The methanol plus 2 ml of chloroform was added to the glass tube and the sample was vortex mixed for 5 min. Then 600 μl of 20 mM acetic acid was added and the two-phase system was vortex mixed for another 10 minutes. After 5 minutes of centrifugation at 1000 g, the lower organic phase was transferred to a new tube and the water phase was washed with 1 ml chloroform. Finally, after 10 minutes of vortex mixing and 5 minutes of centrifugation to induce phase separation, the organic phases were pooled, evaporated and reconstituted in chloroform/methanol (2:1). The lipid extracts were stored at −20 °C until further analysis.

### MTBE method

After transfer of the homogenate to the 15 ml glass tube, the homogenization tube was washed with 1 ml of methanol. The methanol plus 5 ml of MTBE was added to the glass tube and the sample was mixed for 1 h at room temperature. Phase separation was achieved by adding 1.25 ml water. After 5 minutes of mixing the tube was centrifuged at 1000 g for 10 minutes and the upper (organic) phase was collected. The lower phase was washed with a solvent mixture with the same composition as the upper phase and the two organic phases were pooled, evaporated and reconstituted in chloroform/methanol (2:1). The lipid extracts were stored at −20 °C until further analysis.

### Lipid quantification.

Endogenous lipids from mouse liver and heart were detected and quantified using several techniques. FC was quantified using straight-phase HPLC and ELS detection as previously described^10^. Quantification was made against an external calibration curve. This chromatographic set-up was also used to fractionate DG. Quantification of CE, TG, SM, and phospholipids (all from the total extract) and DG (fractionated from the HPLC) was made by direct infusion (shotgun) on a QTRAP 5500 mass spectrometer (Sciex, Concord, Canada) equipped with a robotic nanoflow ion source, TriVersa NanoMate (Advion BioSciences, Ithaca, NJ)^11^. For this analysis, total lipid extracts, stored in chloroform:methanol (2:1), were diluted with internal standard-containing chloroform/methanol (1:2) with 5mM ammonium acetate and then infused directly into the mass spectrometer. The characteristic dehydrocholesterol fragment *m/z* 369.3 was selected for precursor ion scanning of CE in positive ion mode^12^. The analysis of TG and DG was performed in positive ion mode by neutral loss detection of 10 common acyl fragments formed during collision induced dissociation^13^. The PC, LPC and SM were detected using precursor ion scanning of *m/z* 184.1^14^, while the PE, phosphatidylserine (PS), phosphatidylglycerol (PG) and phosphatidylinositol (PI) lipid classes were detected using neutral loss of *m/z* 141.0, *m/z* 185.0, *m/z* 189.0 and *m/z* 277.0 respectively^15^,^16^. For quantification, lipid class-specific internal standards were used. The internal standards were either deuterated or contained diheptadecanoyl (C17:0) fatty acids.

Ceramides (CER), dihydroceramides (DiCER), glucosylceramides (GlcCER) and lactosylceramides (LacCER) were quantified using a QTRAP 5500 mass spectrometer equipped with a Rheos Allegro quaternary ultra-performance pump (Flux Instruments, Basel, Switzerland). Before analysis the total extract was exposed to alkaline hydrolysis (0.1M potassium hydroxide in methanol) to remove phospholipids that could potentially cause ion suppression effects. After hydrolysis the samples were reconstituted in chloroform:methanol:water [3:6:2] and analyzed as previously described^17^.

For the recovery experiments the tissue samples were spiked with non-endogenously present lipids (or endogenous lipids spiked at relatively high levels) and could therefore all be detected by lipid class specific scans using the shotgun approach. In the recovery experiment we therefore also included the PA and phosphatidylcholine plasmalogen (PC P) lipid class, which we could not measure endogenously using our current analytical platform. Due to poor ionization efficiency, FC was derivatized and analyzed as picolinyl esters according to previous publication^18^. See Table 1 for details. With some exceptions, lipids are annotated according to Liebisch *et al*.^19^.

### Statistics

Correlation analysis was performed using the Pearson correlation coefficient. Comparisons between groups were made by one-way ANOVA with Bonferroni correction for multiple testing. Due to the nature of the compositional data with large number of observations that are all expressed as mol% of total amount, and therefore highly correlated with each other, no statistical calculations were made for comparison of lipid species profiles between the three extraction methods.

## Results

### Optimization of tissue homogenization and extraction

The homogenization and extraction steps were optimized using the Precellys 24 and Mixer Mill 301 instruments. We noted that power 5000 was the maximum effect that could be used with the Precellys 24, with the low-cost Sarstedt tubes we normally use, in order to avoid cracks in the tubes. For applications where power 5000 needs to be exceeded, or repeated homogenization cycles at power 5000 required, we recommend equivalent tubes CK28 and CK28-R (reinforced tube walls and caps) provided by Bertin (see discussion).

The time needed for homogenization depended on the tissue type. Muscle, colon and heart tissue needed 2–3 20-second cycles, while liver and brain were sufficiently homogenized already after one 20-second cycle at power 5000.

**Figure 1:**
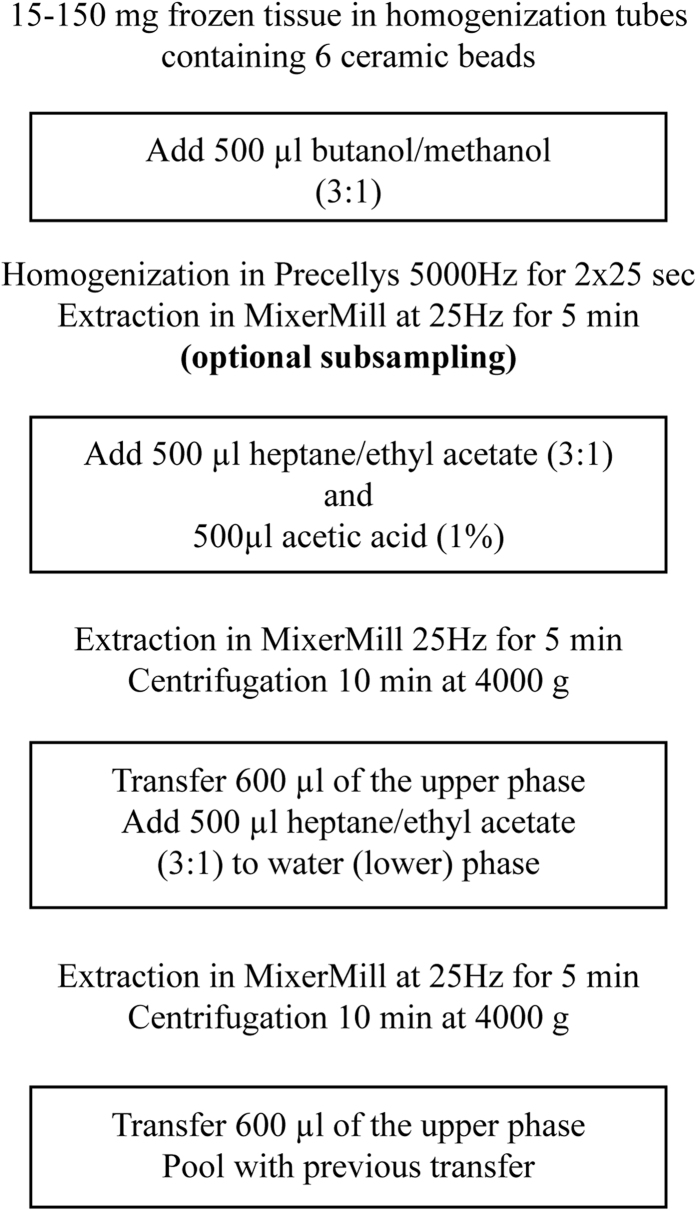
Outline of the BUME extraction protocol for tissue. The steps placed inside the squares can be performed by the robot. After the initial one-phase extraction using the butanol/methanol mixture, the samples can be centrifuged and subsampled for metabolomics studies^28^ or for analysis of more polar phospholipids (see discussion).

The additional time needed in Mixer Mill 301 for sufficient extraction was also investigated and the data showed that 5 min was sufficient and after that no improvement in the recoveries could be observed (data not shown). All transfer steps in the extraction can be made using a pipetting robot. For an outline of the extraction workflow see Fig. 1.

### The BUME method delivers high lipid recoveries

**Figure 2:**
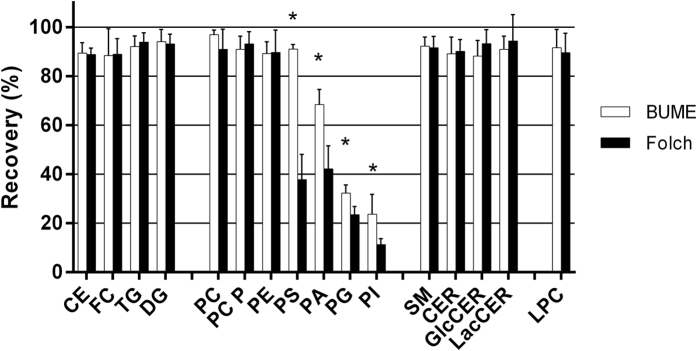
Lipid recoveries. Recoveries of lipids spiked into liver tissue powder show that BUME gives similar or better recoveries then the Folch method. The values are expressed as mean ± SD (n = 6, *P < 0.05 versus Folch).

To determine recoveries, a dog liver matrix spiked with a set of non-endogenous lipids (or endogenous lipids at relatively high levels) was extracted. The lipids in this set were spiked into the samples prior to or after the extraction. The recovery (%) was then calculated as the amount of lipids found in the samples spiked prior to the extraction, divided by the amount of lipids found in the samples spiked after the extraction, multiplied by 100. A second set of non-endogenous lipids were spiked into all samples after the extraction and used for quantification (see also Table 1). As can be seen in Fig. 2 the recoveries were around 90% for most of the investigated lipids and the BUME method performed equally good or better when compared to the Folch method. This is especially true for the acidic phospholipids that show poor recovery using the Folch method.

In order to further verify that lipids do not stick to the plastic tubes and reduce recovery we performed an extraction with radioactive tracers. Radioactive lipids (CE, TG, FC, DG, PC, SM, LPC and a palmitic acid) were spiked into 60 μl of Seronorm Lipid and extracted in plastic tubes according to the method presented here or in glass vials according to previous work^9^. The recoveries were the same for both procedures (Suppl. Fig. 2).

### The BUME method delivers constant recoveries for a wide range of tissue weights

**Figure 3:**
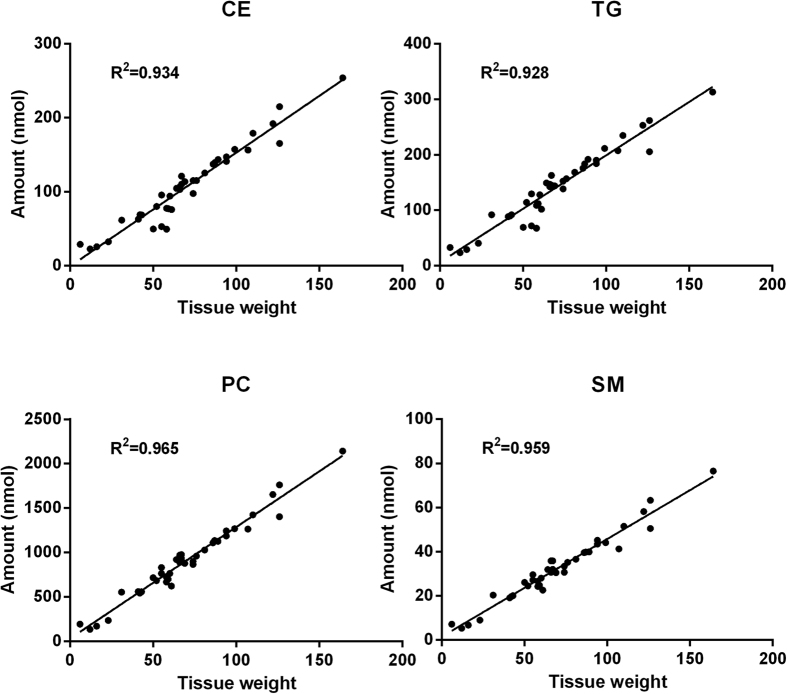
Test of linearity. Different amounts of pulverized liver sample were extracted. The BUME method shows linearity between 15–150 mg tissue for both non-polar (CE and TG) and more polar (PC, SM) lipid classes.

In order to verify that the developed protocol can extract lipids from a wide range of tissue weights, without compromising the recovery, pulverized liver samples (15–150 mg) were added to the Sarstedt tubes and extracted using the developed protocol. The lipids were quantified and the results show linear correlations between the lipid amount and tissue weight for all lipid classes. In Fig. 3, examples are shown for both non-polar (TG and CE) and more polar lipid classes (PC and SM). These results show that the extraction efficiency is not compromised for tissue weights up to 150 mg. The ability to extract 15–150 mg tissue with the same amount of solvent (500 μl BUME mixture for the initial extraction step) makes this method robust and suitable for automation and thereby superior to protocols where the amount of solvent must be adjusted to the sample amount.

### The BUME, Folch and MTBE methods generate similar lipid profiles for most investigated lipid classes

**Figure 4:**
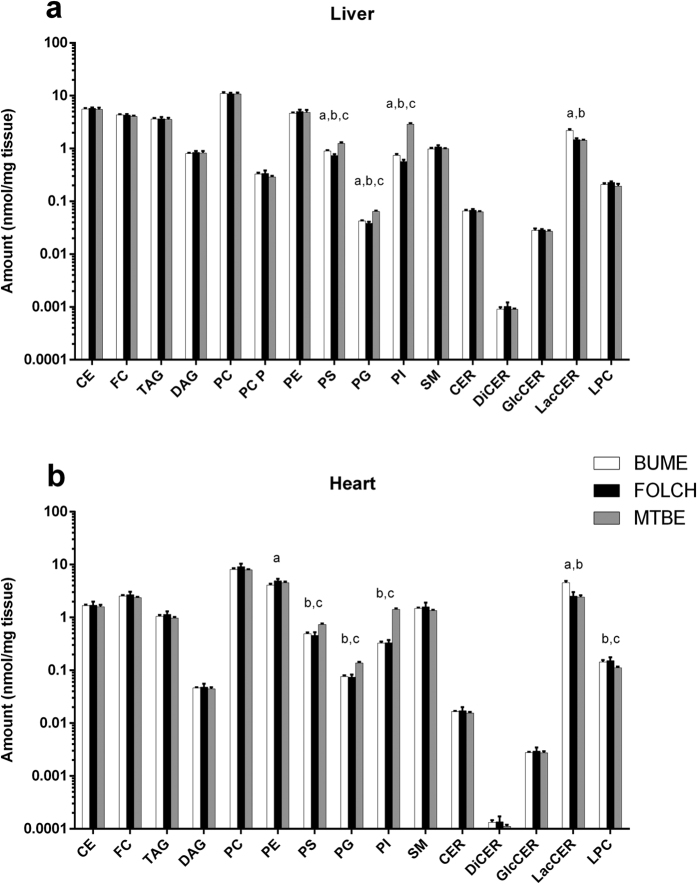
Quantification of lipid classes in liver and heart. The lipid amounts were quantified in mouse liver (**a**) and heart (**b**) after extraction using BUME, Folch or the MTBE method. The data are shown as mean ± SD (n = 6). Significances (p < 0.05) after ANOVA, with Bonferroni correction and post-hoc t-tests, are annotated with a) BUME vs Folch; (**b**) BUME vs MTBE and (**c**) Folch vs MTBE.

**Figure 5:**
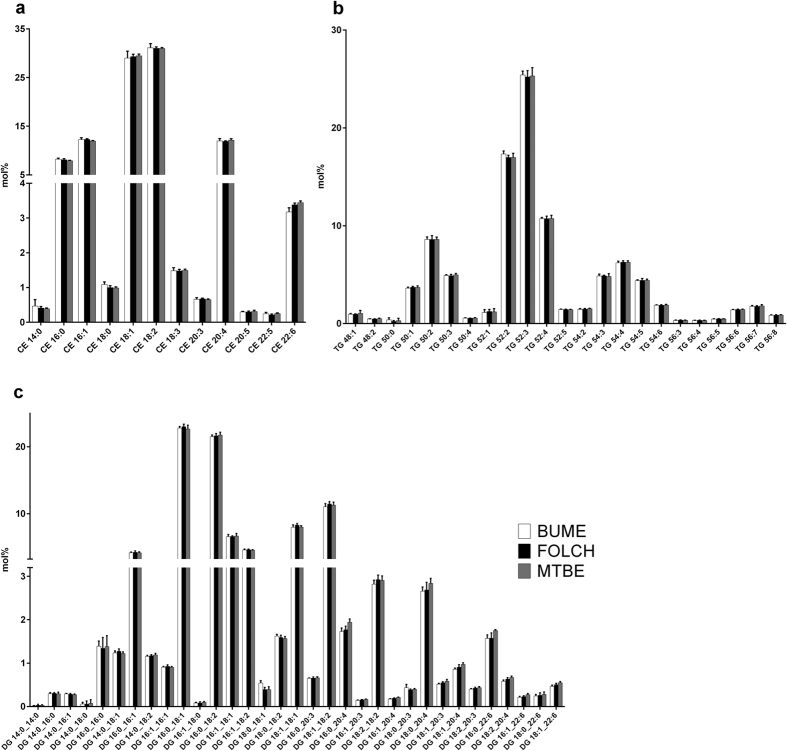
Neutral lipid species profiles. The lipid species profiles of CE (**a**), TG (**b**) and DG (**c**) lipid classes were compared after extraction of liver using the BUME, Folch or MTBE method. For heart profiles see Suppl. Fig. 3. The data are shown as mean ± SD (n = 6).

**Figure 6:**
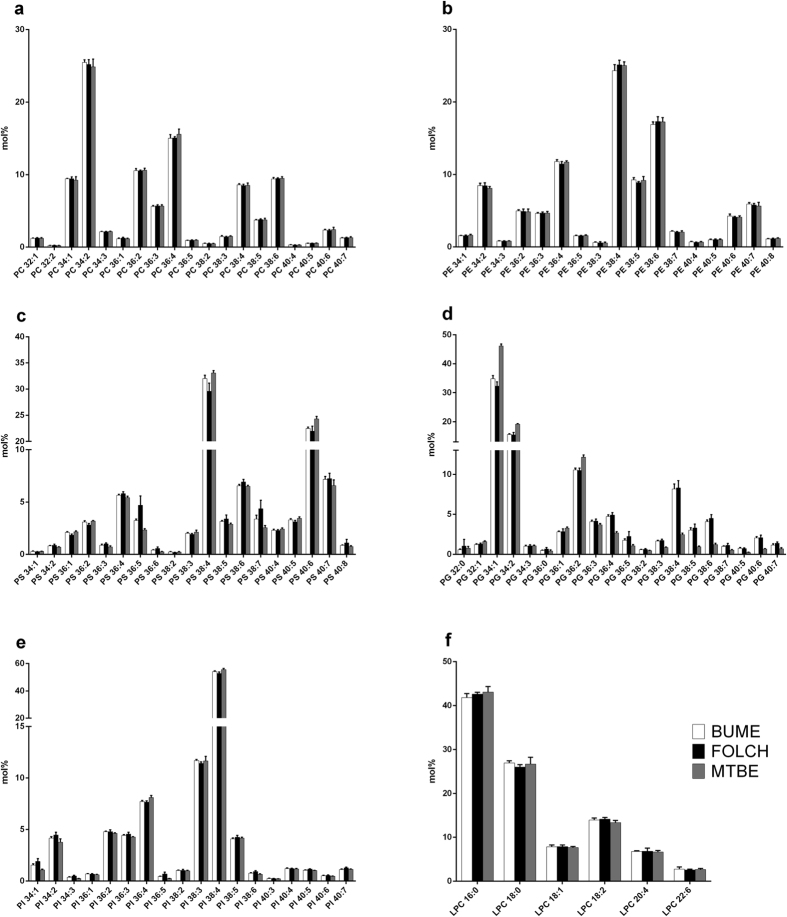
Phospholipid species profiles. The lipid species profiles of PC (**a**), PE (**b**), PS (**c**), PG (**d**), PI (**e**) and LPC (**f**) lipid classes were compared after extraction of liver using the BUME, Folch or MTBE method. For heart profiles see Suppl. Fig. 4. The data are shown as mean ± SD (n = 6).

**Figure 7:**
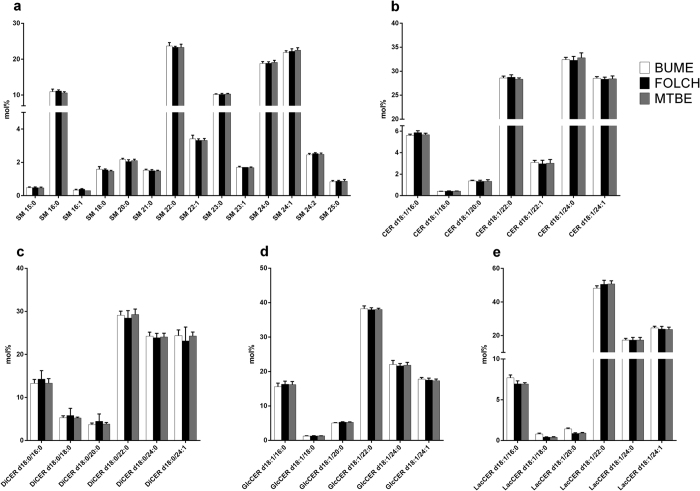
Sphingolipid species profiles. The lipid species profiles of SM (**a**), CER (**b**), DiCER (**c**), GlcCER (**d**), and LacCER (**e**) lipid classes were compared after extraction of liver using the BUME, Folch or MTBE method. For heart profiles see Suppl. Fig. 5. The data are shown as mean ± SD (n = 6).

For further validation, the BUME method was compared to the Folch and MTBE methods^8^. For this both liver and heart were extracted using the three methods and lipid class amounts and lipid species profiles were compared. The results showed that all three methods gave similar lipid class amounts in both liver and heart (Fig. 4). The main differences between the methods were observed for the negatively charged phospholipids PS, PI and PG, where MTBE showed higher levels compared to both Folch and BUME methods. However, the LacCER lipid class were extracted with higher recovery using the BUME method as compare to both Folch and MTBE. The lipid species profiles were also similar between the three methods (Figs 5, 6, 7 for liver and Suppl. Figs 3–5 for heart). Again the main differences were observed for the negatively charged phospholipids and the LacCER.

## Discussion

In the developing era of lipidomics new demands are put on the analytical and sample preparation techniques. While there have been publications describing the automated extraction of fluids, automated and miniaturized sample preparation techniques for tissue are less common. In this paper we describe a simple and fast, chloroform-free method for total lipid extraction of animal tissue. The BUME method presented here shows high recoveries and can be used for a wide range of tissue weights. The method, which combines sample collection, storage, homogenization and extraction in one single tube, is performed in a small 2-ml polypropylene tube and solvent transfers can therefore be automated using a 96-well pipetting robot. Automation substantially reduced manual work with repeated aspiration and dispensing steps. In fact, less than 50% total lab time (less than 25% manual time) was required to process a 24-sample batch as compared to the Folch and MTBE methods. In addition, the solvent consumption was substantially lower as compared to Folch (50% reduction) and MTBE (75% reduction).

Tissue homogenization is a prerequisite for optimal lipid extraction and is often performed manually, one sample at a time. In this paper we use homogenization tubes with ceramic beads for automated tissue homogenization. There are several types of homogenization tubes and beads available from instrument manufacturers and after evaluation we concluded that 2 ml propylene tubes in combination with 3-mm ceramic beads could homogenize all tested tissues at power 5000. At higher speeds, cracks could be observed in the low-cost Sarstedt tubes (not designed for homogenization), especially when homogenizing small tissue pieces in less than 500 μl BUME solvent. Therefore, for applications requiring higher power or smaller solvent volumes and/or samples weights, tubes originally optimized for homogenization (Bertin CK28) or even reinforced tubes (Bertin CK28-R) should be used.

The use of plastics in combination with lipids and organic solvents may not be ideal. However, polypropylene tubes have been used previously for lipid extraction^4^,^20^,^21^ and our tests with radiolabelled tracers show recoveries identical to what we observed using glass vials, indicating that lipids do not adhere to the plastic. However, glass should be used for long time storage of the final lipid extract.

The use of 2 ml homogenization tubes enables us to fit 24-samples in a 96-well format. In practice this means that custom-made tube holders were made with holes for the sample tubes at every second position on every second row on an imaginary 96-well plate (Suppl. Fig. 1). During the initial stages of the sample preparation, the tubes are stored in alumina blocks kept at about −20 °C (racks kept in contact with dry ice or kept on Styrofoam insulation while processing a batch of samples), which enables us to keep the tissues frozen from sampling through homogenization and completed single-phase extraction. This is important since higher temperatures might lead to artificial formation of lipids such as LPC from PC and DG from TG. The set up presented here can also be used for the analysis of other fragile lipids such as short-chain acyl-CoA, which is rapidly degraded in thawing tissues. As could be expected, small acyl-CoA are however not efficiently extracted using the BUME method but require dedicated extraction protocols^22^.

Even though automation of chloroform-based methods has been developed^21^, the drawbacks with using chlorinated solvents and a lower lipid-containing organic phase led us to search for alternative methods. There are several solvent systems that can be used in order to create a lipid-containing upper phase. Traditionally, isopropanol has been used together with hexane^23^ or heptane^24^. However, these methods have been shown to have limited extraction efficiencies for polar lipids. Recently Matyash *et al*. published a method based on MTBE which also results in a lipid-containing upper phase^8^. Using this method for lipid extraction of plasma and tissue, the authors show that the procedure is associated with high recoveries of a wide range of plasma lipids and fully comparable with the Folch method. Further development by Surma *et al*., with some modifications in initial sample to solvent ratio, enables the method to be scaled down and automated extraction of plasma lipids could be performed in the 96-well format^25^. Whether this method could be applied to tissue needs to be further investigated and lies outside the scope of this study.

The use of butanol has been described previously and shows good extraction properties, especially for the more polar lipids^26^,^27^. As described more in detail in our previous work, the BUME system has a low and flexible solvent to sample ratio, allowing up to 150 mg tissue to be homogenized and extracted in only 500 μl BUME mixture. Furthermore, the low total solvent volume (1.5 ml) added is beneficial since it allows the complete process, from sampling to final extraction, to be performed in a single 2 ml tube. This in turn allows a rapid, reproducible and automated process. Even though the automation of the BUME method is beneficial, manual liquid handling works equally well as long as the samples are kept frozen until the homogenization in cold BUME mixture is completed. Also, the extraction steps using Mixer Mill 301 may be replaced by vortex mixing. However, the mixing time should be extended to 10 minutes since vortex mixing is not as efficient.

The centrifugation of the lipid extracts is preferably performed in a swing out rotor (as opposed to a fixed angle rotor) since it will generate a flat intermedia phase containing the debris and precipitate. This will in turn increase the precision of the pipetting and reduce the chance of contaminants in the lipid extract. We have observed that for certain combinations of tissue types and sample sizes, emulsions can be formed between the lipid extract and the buffer phase that are difficult to brake with the standard centrifugation process of 4000 rpm for 10 minutes with a swing-out plate centrifuge. Stronger centrifugation conditions and longer time solved this rare problem. A challenge when working with tissue, as compared to biofluids, is to find a homogenous sample material. In this study we used a pool of pulverized dog livers for validating both recovery and linearity of the method. For the recovery experiments, the liver was used as a matrix to which we spiked our standards. The use of a relevant matrix is important when investigating recoveries since possible lipid-matrix interactions need to be determined.

From the linearity results we can see that the correlations between lipid amounts and tissue weights are associated with some variation. The reason for this can be sustained heterogeneity in the sample material or inaccurate weighing. Moreover, in the experiment we also added the internal standards after the extraction in order to clearly see any non-linearity. Therefore, the analytical variation will be reduced when internal standards are added before the extraction. An indication that the variation is related to systematic errors such as weighing or pipetting, rather than variation in the extraction or the analysis, is that the relative distribution of lipids is maintained. For example, if the PC level is low, so are the PE and SM values (Suppl. Fig. 6). When expressed in this way, the results show excellent reproducibility over the whole range of tissue amounts.

The extraction of endogenous lipids using BUME, Folch and MTBE method showed that all investigated lipid classes were extracted with similar or better recoveries using the BUME method as compared to Folch. However the results also showed that the polar and negatively charged phospholipids PI, PG and PS were more efficiently extracted with the MTBE method as compared to both BUME and Folch. The main reason for this is probably the relative high polarity of the MTBE phase and the large solvent volume used. One drawback with the high polarity of the MTBE phase is that the extracts will contain more polar metabolites that, depending on method, might compromise the analysis. Furthermore, the high levels of water in the MTBE phase will also affect evaporation of the extracts which in our hands turned out to be very time consuming.

From the data described above we conclude that the two-phase system generated in BUME (and also in Folch and probably also in MTBE) is not suited for the extraction of negatively charged phospholipids. Instead these lipids should be extracted with a one-phase system. As previously shown^28^, a butanol:methanol mixture has great potential in extracting these lipids and we therefore believe that by removing an aliquot from the initial one-phase system in the BUME method (Fig. 1), these lipid classes could be attained with high recovery.

In conclusion, the new BUME methods for biofluids and tissue samples presented here are superior to the old, laborious and toxic, but still commonly used chloroform-based extraction methods. The use of the BUME mixture as the single-phase extraction solvent enables us to collect the sample at the site of the experiment, rapidly snap-freeze the sample and perform both the automated homogenization and extraction in a single 2-ml homogenization tube with the tissue sample frozen at all time to prevent biochemical degradation of lipids until total lipid extraction is completed. Furthermore, the method shows high recoveries for a wide range of lipids and lipid species profiles are comparable with the gold standard Folch method.

## Additional Information

**How to cite this article**: Löfgren, L. *et al*. The BUME method: a new rapid and simple chloroform-free method for total lipid extraction of animal tissue. *Sci. Rep.* **6**, 27688; doi: 10.1038/srep27688 (2016).

## Supplementary Material

Supplementary Information

## Figures and Tables

**Table 1:** Standards and MS methods used for the recovery experiment.

**Standard 1**^a^	**Standard 2**^a^	**MS method**^b^
CE 17:0	d_5_-CE 18:0	+PIS 369 (CE 17:0) +PIS 375 (CE d5 18:0)
d_7_-FC	–	UPLC-MS/MS^18^
TG 17:0/17:0/17/0	d_6_-TG 16:0/16:0/16:0	+NL 273.2 (C16:0) +NL 287.2 (C17:0)
DG 14:0/14:0/0:0	DG 17:0/17:0/0:0	+NL 245.2 (C14:0) +NL 287.2
PC 17:0/17:0	PC 17:0/20:4	−PIS 269.2 (C17:0) −PIS 303.2 (C20:4)
PC P-18:0/18:1^c^	PC P-18:0/20:4^c^	+PIS 184.1
PE 17:0/17:0	PE 17:0/20:4	−PIS 269.2 −PIS 303.2
PS 17:0/17:0	PS 17:0/20:4	−PIS 269.2 −PIS 303.2
PA 17:0/17:0	PA 17:0/20:4	−PIS 269.2 −PIS 303.2
PG 17:0/17:0	PG 17:0/20:4	−PIS 269.2 −PIS 303.2
PI 18:0/18:0^c^	PI 17:0/20:4	−PIS 269.2 −PIS 283.2 (C18:0) −PIS 303.2
SM d18:1/12:0	SM d18:1/17:0	+PIS 184.1
CER d18:1/18:0^c^	CER d18:1/17:0	+PIS 264.2
GlcCER d18:1/16:0^c^	GlcCER d18:1/12:0	+PIS 264.2
LacCER d18:1/12:0	GlcCER d18:1/12:0	+PIS 264.2
LPC 17:0	PC 17:0/20:4	+PIS 184.1

^a^Standard 1 was added before, and standard 2 after the extraction. As a reference (100% recovery) both standards were added after the extraction.

^b^With the exception of free cholesterol, which doesn’t ionize using electrospray, the lipids were measured using precursor ion scanning (PIS) and neutral loss (NL) experiment during a constant nano-flow infusion. See methods for further information.

^c^These lipids were added in high amounts so that the contribution of the endogenously present lipid could be neglected.
