# Supplementary material for: The BUME method: a new rapid and simple chloroform-free method for total lipid extraction of animal tissue
Source: Sci Rep. 2016 Jun 10;6:27688. doi: 10.1038/srep27688 (PMC4901324; doi:10.1038/srep27688)
Supplement: Supplementary Information [file srep27688-s1.pdf]

Supplementary information

The BUME method: a new rapid and simple chloroform-free method for total lipid extraction of animal tissue

Lars Löfgren, Gun-Britt Forsberg, and Marcus Ståhlman

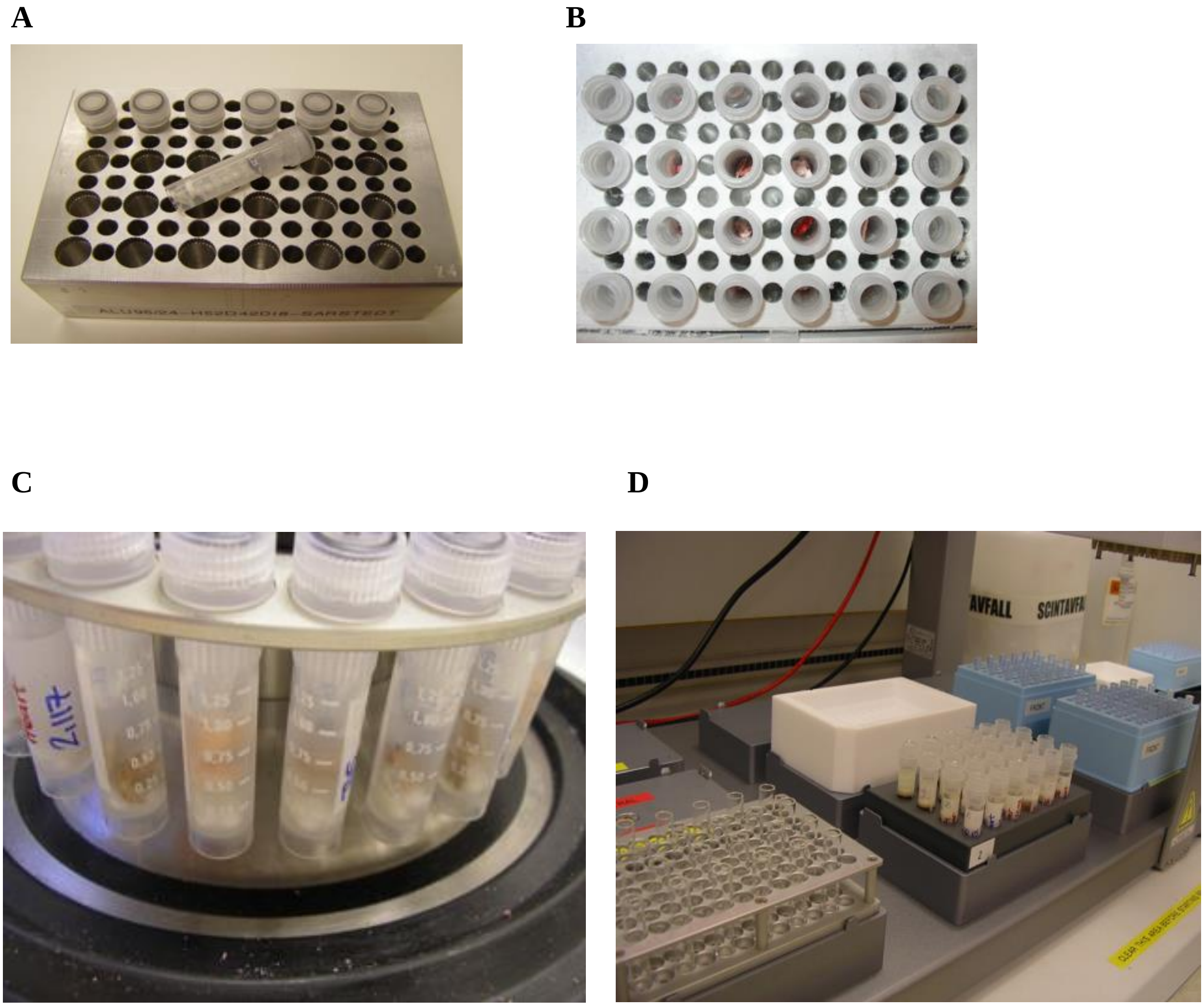

Suppl. Fig 1. Sample processing and automated lipid extract handling with the BUME method. Panel A and B shows custom made alumina blocks for cold handling during sample preparation. Panel C shows homogenization tubes placed in the Precellys24 instrument. Panel D shows robotic setup for automated transfer of solvents and lipid extract.

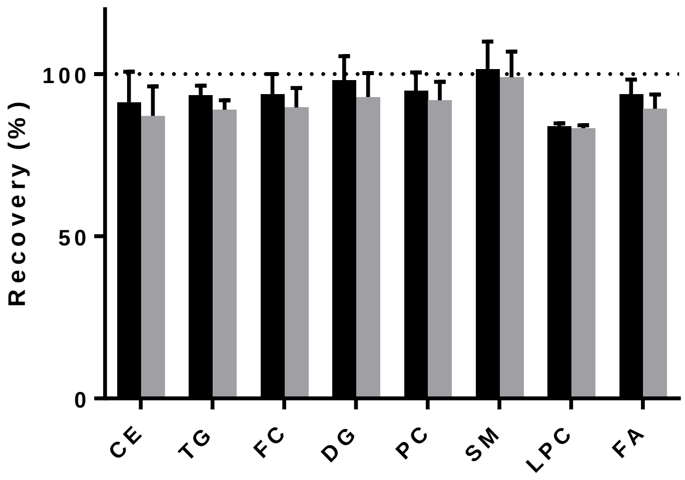

Suppl. Fig 2. Recovery of radioactive lipids spiked into 60µl Seronorm Lipid and extracted using the BUME method. The extraction was either made in plastic tubes (grey bars) as described in this study or in glass vials (black bars) according to previous work[1]. The data (n=4) are shown as mean ± SD. The glass vial protocol includes three automated extraction steps while the present protocol for tissue includes two extraction steps that may explain generally slightly higher recoveries with the glass vials.

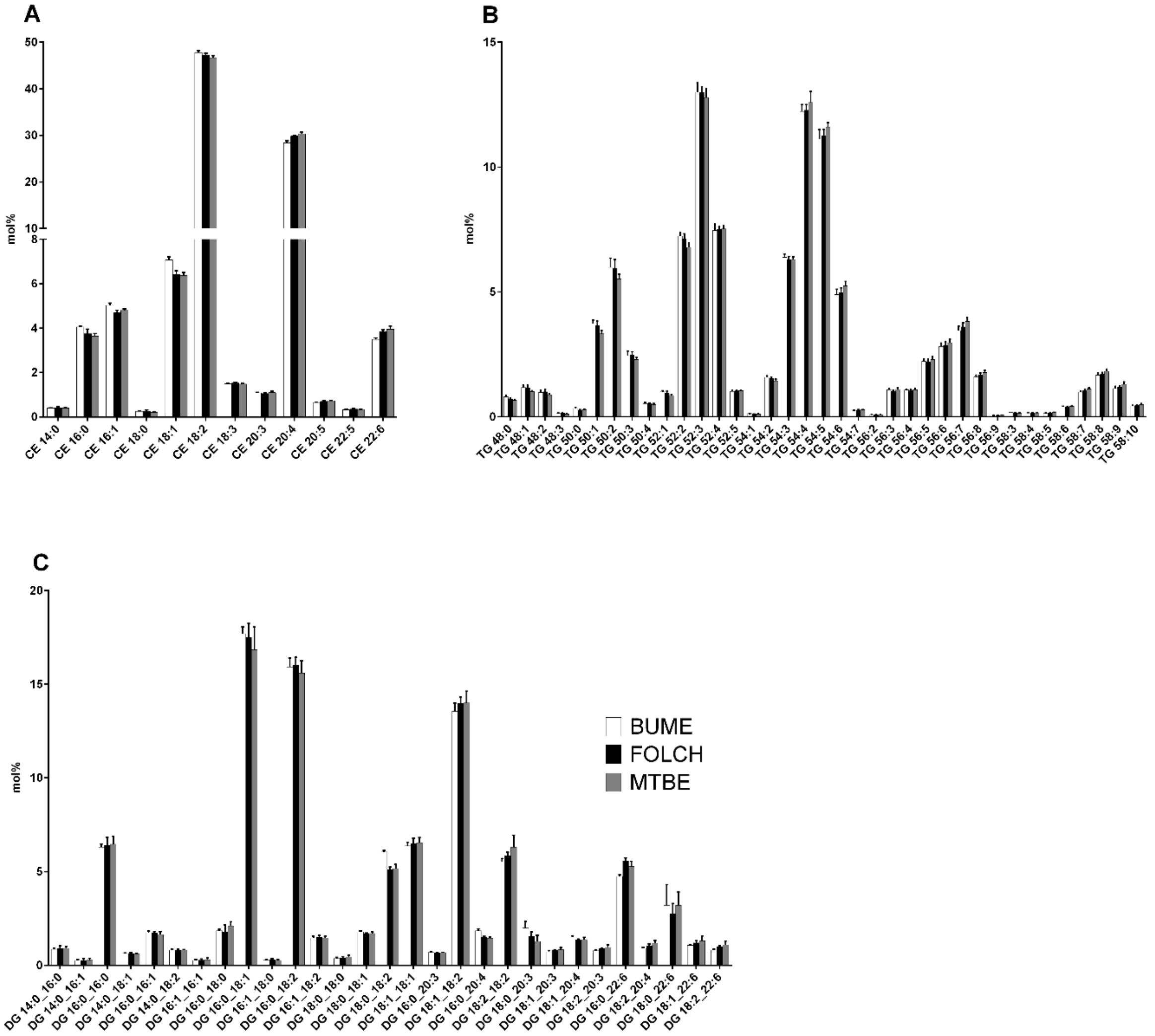

Suppl. Fig 3. Neutral lipid species profiles. The lipid species profiles of CE (**a**), TG (**b**) and DG (**c**) lipid classes were compared after extraction of heart using the BUME, Folch or MTBE method. The data are shown as mean ± SD (n=6).

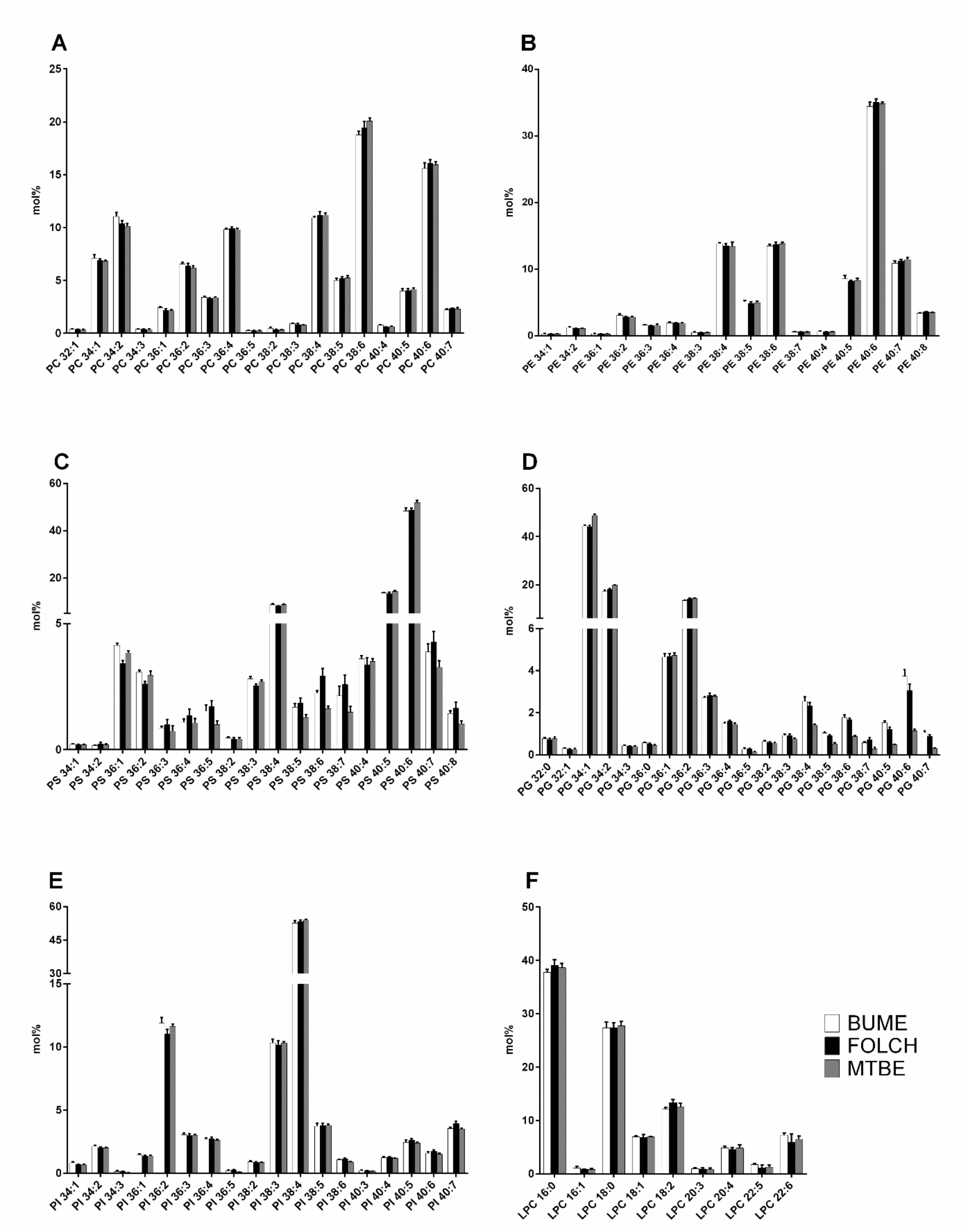

Suppl. Fig 4. Phospholipid species profiles. The lipid species profiles of PC (**a**), PE (**b**), PS (**c**), PG (**d**), PI (**e**) and LPC (**f**) lipid classes were compared after extraction of heart using the BUME, Folch or MTBE method. The data are shown as mean ± SD (n=6)

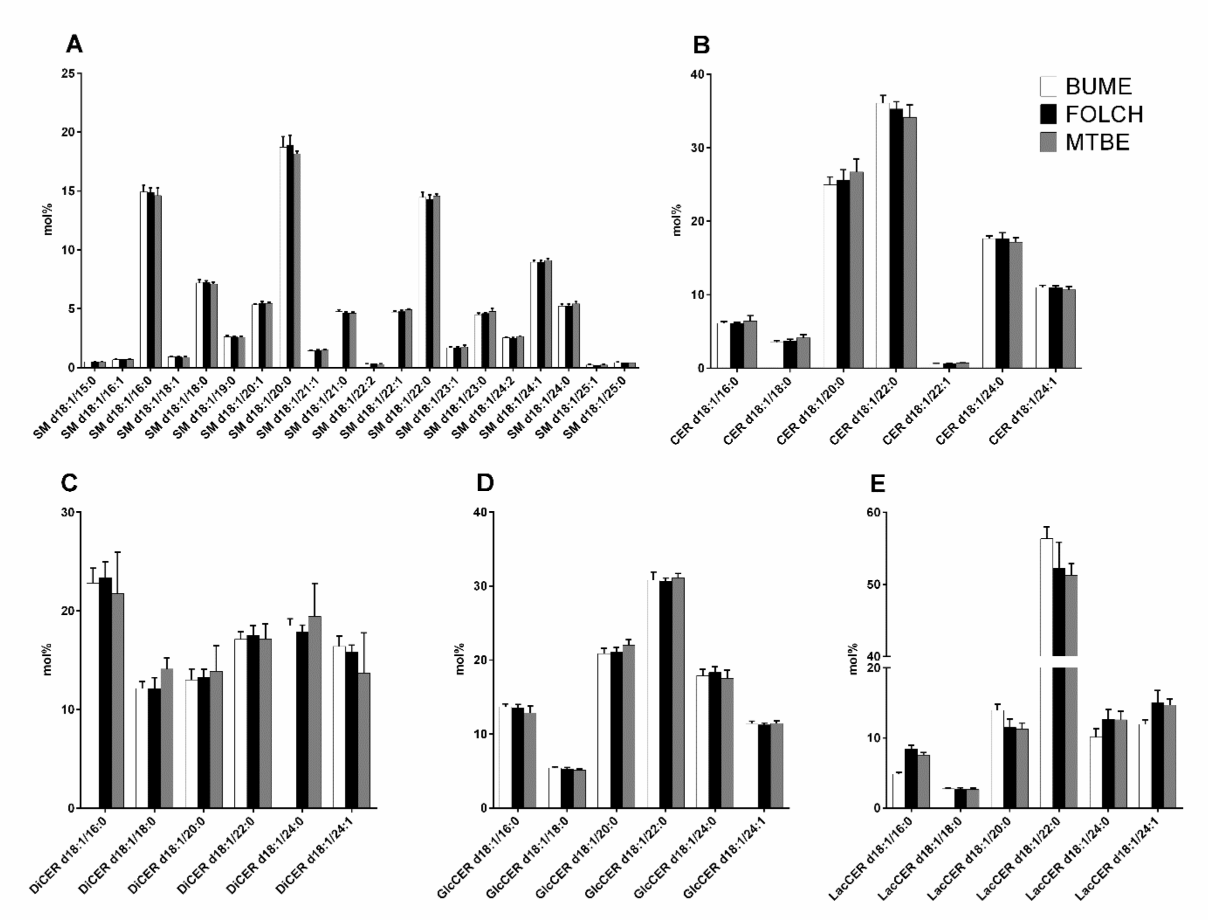

Suppl. Fig 5. Sphingolipid species profiles. The lipid species profiles of SM (**a**), CER (**b**), DiCER (**c**), GlcCER (**d**), and LacCER (**e**) lipid classes were compared after extraction of heart using the BUME, Folch or MTBE method. The data are shown as mean ± SD (n=6)

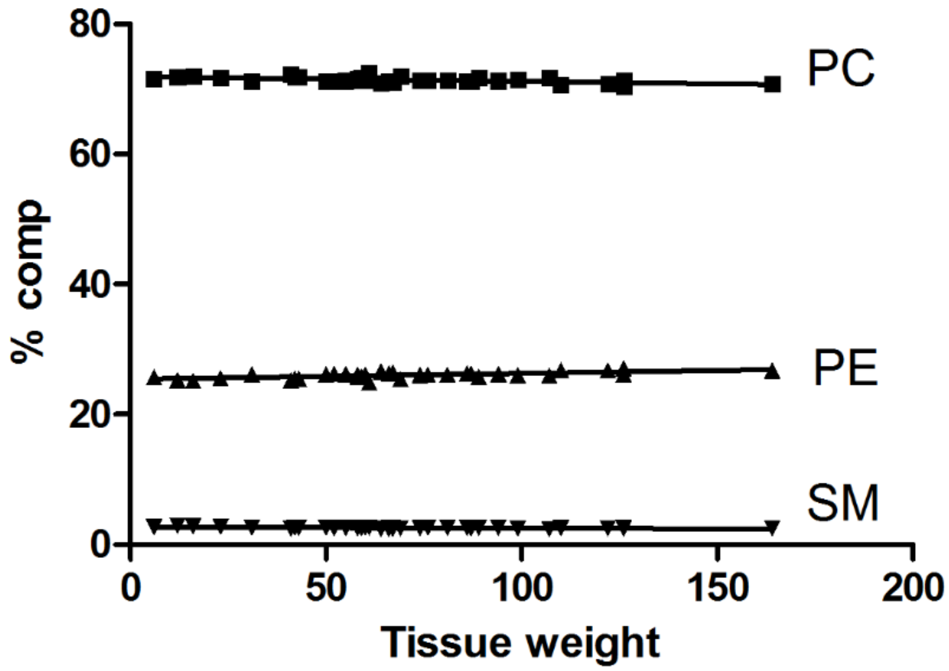

Suppl. Fig 6. Relative composition of PC, PE and SM in 15-150 mg liver tissue extracted with the BUME method. Despite some variation in the linearity experiment (Fig. 3), the composition of lipids were stable and not affected by the tissue weight.

REFERENCES

1 Lofgren, L. *et al.* The BUME method: a novel automated chloroform-free 96-well total lipid extraction method for blood plasma. *Journal of lipid research* **53**, 1690-1700 (2012).
